# Application value of joint STOP-Bang questionnaire and Epworth Sleepiness Scale in screening for obstructive sleep apnea

**DOI:** 10.3389/fpubh.2022.950585

**Published:** 2022-09-29

**Authors:** Zhenzhen Zheng, Yitao Zhang, Mingdi Chen, Xiaojuan Chen, Chunhe Li, Chaoyu Wang, Jinru Zhu, Junyan Lin, Xudong Ou, Zhihong Zou, Zhiwei Wang, Junzhong Deng, Riken Chen

**Affiliations:** ^1^The Second Affiliated Hospital of Guangdong Medical University, Zhanjiang, China; ^2^Yangjiang Hospital of Traditional Chinese Medicine, Yangjiang, China; ^3^Medical College of Jiaying University, Meizhou, China; ^4^The First Affiliated Hospital of Guangzhou University of Chinese Medicine, Guangzhou, China; ^5^Taishan Hospital of Traditional Chinese Medicine, Jiangmen, China; ^6^State Key Laboratory of Respiratory Disease, National Clinical Research Center for Respiratory Disease, Guangzhou Institute of Respiratory Health, The First Affiliated Hospital of Guangzhou Medical University, Guangzhou Medical University, Guangzhou, China

**Keywords:** obstructive sleep apnea, Epworth Sleepiness Scale, STOP-Bang questionnaire, diagnostic, polysomnography

## Abstract

**Objective:**

This paper evaluates the application value of the STOP-Bang questionnaire combined with the Epworth Sleepiness Scale (ESS) in screening for obstructive sleep apnea (OSA) in the population.

**Method:**

Thousand-six hundred seventy-one patients with suspected OSA who visited the Sleep Medicine Center of the First Affiliated Hospital of Guangzhou Medical University from August 2017 to August 2020 were monitored by overnight polysomnography (PSG) after completing the ESS scale and STOP-Bang questionnaire. The sensitivity, specificity, positive predictive value, negative predictive value and receiver operating characteristic (ROC) curves of the two scales were calculated, and the accuracy in predicting OSA of the STOP-Bang questionnaire combined with ESS was analyzed.

**Results:**

With Apnea Hypopnea Index (AHI) cutoffs of ≥5, ≥15 and ≥30 events/h, the areas under the ROC curve scored by STOP-Bang were 0.724, 0.703 and 0.712, and those of ESS were 0.632, 0.634 and 0.695; the diagnostic odds ratio (DOR) values of STOP-Bang for OSA, moderate to severe OSA, and severe OSA were 3.349, 2.651 and 3.189, and those of ESS were 2.665, 2.279 and 3.289. The STOP-Bang score of three was used as the cut-off point for OSA diagnosis with higher sensitivity and lower specificity, while ESS had higher specificity. STOP-Bang (≥3) combined with ESS significantly improved its specificity for predicting OSA.

**Conclusion:**

The STOP-Bang questionnaire is a simple and effective new tool for screening patients for OSA, while a STOP-Bang score of ≥3 combined with ESS can further improve its specificity. Thus, we suggest further screening with ESS after a STOP-Bang score of ≥3 in suspected patients.

## Introduction

Obstructive sleep apnea (OSA) is a recurring narrowing or partial or complete collapse of the airway, snoring/apnea and low ventilation during sleep, leading to frequent hypoxemia, hypercapnia and common sleep-related breathing disorders. And as we know the Editorial Board of The AASM Manual for the Scoring of Sleep and Associated Events: Rules, Terminology and Technical Specifications (AASM Scoring Manual) would like to notify the membership and the sleep community that an update for the AASM Scoring Manual (Version 2.4) was released April 1, 2017 (1). The prevalence of OSA is about 1–5% in children, 9% in adult women and 24% in adult males (2, 3). OSA is harmful, can even lead to death and is associated with the increased mortality of patients (4, 5). Although the connection remains debated, several mechanisms such as intermittent hypoxemia, sleep deprivation, hypercapnia disruption of the hypothalamic-pituitary-adrenal axis have been associated with poor neurocognitive performance. Different treatments have been proposed to treat OSAS patients as continuous positive airway pressure (CPAP), mandibular advancement devices (MAD), surgery; however, the effect on neurocognitive functions is still debated. CPAP treatment seems to improve cognitive defects associated with OSA. Limited studies have evaluated the effects of the other therapies on cognitive function as oral appliance or barbed surgery (6). The scary thing is that a lot of OSA in the population goes undiagnosed (7, 8). As we all know, polysomnography (PSG) is the gold standard for the diagnosis of OSA, but it is difficult to apply PSG widely in primary hospitals as sleep rooms and professional and technical personnel are required, while PSG examination is expensive, complicated and time-consuming. The STOP-Bang questionnaire is a simple and effective screening tool for the risk assessment of suspected sleep disordered breathing (9–11) which includes eight indicators: snoring (S), tiredness (T), observed apnea during sleep (O), blood pressure (P), BMI, age, neck circumference and gender; scores ≥3 are 93% sensitive and 43% specific for moderate OSA, and 100% sensitive and 37% specific for severe OSA (12). It is not difficult to see that although the STOP-Bang questionnaire has good sensitivity, its specificity is poor. A meta-analysis confirms the high performance of the STOP-Bang questionnaire in the sleep clinic and surgical population for screening of OSA. The higher the STOP-Bang score, the greater is the probability of moderate-to-severe OSA (13). It was recently found that STOP-Bang combined with serum bicarbonate can significantly improve the diagnostic value of STOP-Bang for OSA patients (14). Considering that blood drawing tests are troublesome and time-consuming, while the Epworth Sleepiness Scale (ESS) is relatively easy to perform, this study evaluated STOP-Bang and ESS for suspected OSA patients, and then the statistical analysis of the PSG data was completed to further evaluate the application value of STOP-Bang combined with ESS for screening for OSA in the population. We hypothesized that the combination of ESS would significantly improve the specificity of STOP-Bang in predicting OSA.

## Materials and methods

### Study subjects

All participants were recruited from the Sleep Medical Center of the First Affiliated Hospital of Guangzhou Medical University, Guangzhou, China, from August 2017 to August 2020. From a total of 1,861 patients, 1,671 were eventually included: 1,300 males and 371 females; mean age of 47.45 ± 13.90 years; average neck circumference of 38.36 ± 3.93 cm; and mean BMI of 26.49 ± 4.20 kg/m^2^. This study was approved by the Ethics Committee of the First Affiliated Hospital of Guangzhou Medical University with Ethical Approval No. 05, 2017, and all patients gave and signed their informed consent. The inclusion criteria were: (1) older than 18 years; (2) total sleep time of >4 h; (3) autonomous behavior and cognitive ability; and (4) able to answer the questionnaire. The exclusion criteria were: (1) history of various mental and psychological diseases; (2) brain tumors or epilepsy; (3) long-term or current use of benzodiazepines, barbiturates or other sedative and sleeping drugs; (4) severe organ failure leading to an inability to complete the examination; (5) previously diagnosed or treated; (6) did not complete the questionnaire; (7) total sleep time of < 4 h; and (8) OSA dominated by central or mixed events.

### Methods

In our study, we collected the basic data of the 1,671 suspected patients: (1) basic anthropological data; (2) basic demographics (e.g., gender, age, occupation); (3) anthropometric parameters (height, weight, neck circumference, waist circumference, etc.); (4) previous history (history of hypertension, diabetes, cardiovascular and cerebrovascular diseases, and other related diseases); (5) personal history (smoking and drinking); and (6) sleep-related breathing events (e.g., snoring, apnea, sleep suppression). Patients were asked to complete ESS and STOP-Bang 1 h before the PSG examination. According to the PSG monitoring results, the patients were divided into the normal group [AHI < 5 events/h (*n* = 470)], mild OSA group (AHI ≥5 and < 15 events/h [*n* = 378)], moderate OSA group [AHI ≥ 15 and < 30 events/h (*n* = 320)] and severe OSAHS group [AHI ≥30 events/h (*n* = 503)].

### Questionnaire

The STOP-Bang questionnaire has eight questions on snoring, tiredness, observed apnea, hypertension, body mass index >35 kg/m^2^, age >50, neck circumference >40 cm and male that are answered with “yes” or “no.” It adds one point for “yes” and zero points for “no.” When the total points are >3, this indicates that the patient is at high risk for OSA. ESS, which includes 8 questions, asks respondents to rate their sleepiness from zero to three in eight daily situations. For each question, a score of zero indicates no lethargy, and one, two and three indicate light, moderate and heavy lethargy respectively. The highest score of ESS is 24 (the most excessive daytime sleepiness), with a threshold for daytime sleepiness of 10 points or more.

### Polysomnography

All patients were synchronously monitored with an Alice 5 PSG (Philips Wellcome, USA) for at least 7 h, and the use of alcohol, coffee, sedatives and hypnotics was prohibited on the same day. The monitoring indicators included electro-encephalogram, electromyography, blood oxygen saturation, electro-oculogram, electrocardiogram, snoring, mouth airflow, nasal airflow, chest breathing and body position. The raw data was automatically read by the instrument, then manually analyzed by trained sleep professionals for parameters such as sleep duration and sleep breathing events based on the *Manual for the Scoring of Sleep and Associated Events* published by the American Academy of Sleep Medicine (AASM) in 2012, and finally corrected by the same physicians (15). According to the guidelines for the diagnosis and treatment of OSA, patients were defined as having OSA when their obstructive apnea was dominated by respiratory events and their Apnea Hypopnea Index (AHI) was not below five events/h. Patients with suspected OSA were classified into four groups based on AHI: AHI < 5 events/h, AHI ≥ 5 and < 15 events/h, AHI ≥ 15 and < 30 events/h and AHI ≥ 30 events/h.

### Sample size calculation

We take all the data into study and the sample size was not calculated.

#### Reduce the potential for bias

Firstly, it was not the same person who conducted the questionnaire and the PSG and they don't know the patient's condition. Secondly, the sample size is very huge in our study. Lastly, there are fewer interference factors in our study because both the questionnaire and PSG data were obtained from the same patient.

### Statistical analysis

Statistical analysis was performed using SPSS v16.0. One-Way ANOVA was adopted for the normal distribution of data. *Post-hoc* analysis was conducted for comparison between the two groups. The chi-square test was used for comparison between count data groups. The diagnostic results of each scale and PSG were calculated as the sensitivity, specificity, positive predictive value and negative predictive value of each scale in a four-grid scale form. The diagnostic results of PSG and each scale were analyzed in a four-fold table, and the sensitivity, specificity, positive predictive value and negative predictive value of each amount was calculated. The ROC curves were used to analyze the OSA diagnostic performance of STOP-Bang combined with ESS.

## Results

### General data

**Figure 1:**
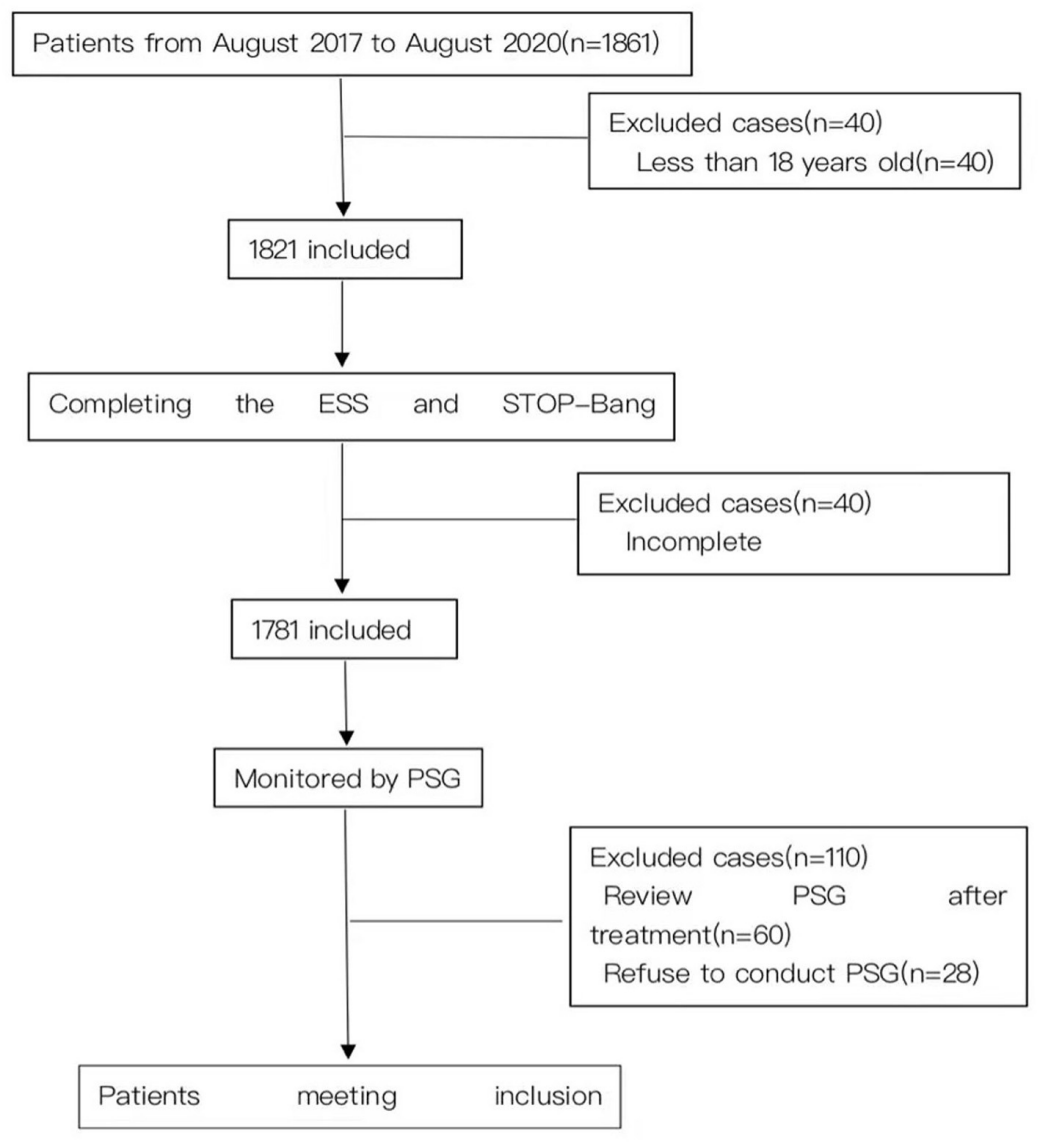
Flow diagram.

A total of 1,671 suspected patients (including 1,300 males) were recruited for this study (Figure 1). The mean age of the subjects was 47.45 ± 13.90 years old, the mean BMI was 26.49 ± 4.20 kg/m^2^ and the mean neck and waistline circumferences were 38.36 ± 3.93 and 95.37 ± 13.90 cm respectively. The mean AHI of the subjects was 26.64 ± 27.69 events/h, and the mean lowest oxygen saturation (LSpO2) was 77.29 ± 14.60%. The mean ESS and STOP-Bang scores were 8.12 ± 5.79 and 3.54 ± 1.50 respectively. There were no statistically significant differences in age and ESS scores between the mild and moderate OSA groups. In addition, there were statistically significant differences in other items among the four groups. The proportion of males in the mild group was higher than in the normal group, higher in the moderate group than the mild group, and higher in the severe group than the moderate group, and the differences were statistically significant (*P* < 0.05). Similarly, these differences were reflected in the indicators of BMI, neck circumference, waist circumference, AHI, ESS and STOP-Bang (Table 1).

**Table 1:** Baseline characteristics of study subjects.

	**All**	**AHI < 5**	**5 ≤ AHI < 15**	**15 ≤ AHI < 30**	**AHI≥30**	**F/χ2**	***P***
*n*	1,671	470	378	320	503		
Male (*n*, %)	1,300	306	282	253	459	99.075	< 0.001
Age (years)	47.45 ± 13.90	47.51 ± 15.05	49.60 ± 13.41	49.76 ± 13.96	44.33 ± 12.49	14.748	< 0.001
BMI (kg/m^2^)	26.49 ± 4.20	24.64 ± 4.06	25.92 ± 3.42	26.45 ± 3.74	28.65 ± 4.18	88.288	< 0.001
NC (cm)	38.36 ± 3.93	36.17 ± 3.82	37.90 ± 3.43	38.48 ± 3.26	40.69 ± 3.47	136.057	< 0.001
WC (cm)	95.37 ± 13.90	89.34 ± 11.17	93.80 ± 9.85	95.47 ± 9.78	102.11 ± 17.64	80.49	< 0.001
AHI (events/h)	26.64 ± 27.69	1.64 ± 1.62	9.26 ± 2.99	20.56 ± 3.55	65.60 ± 13.30	6,751.75	< 0.001
MinSPO_2_	77.29 ± 14.60	88.62 ± 6.11	82.73 ± 9.15	78.23 ± 8.58	62.04 ± 13.72	628.22	< 0.001
ESS	8.12 ± 5.79	6.27 ± 5.20	7.36 ± 5.23	7.26 ± 5.27	10.98 ± 5.99	68.702	< 0.001
STOP-Bang	3.54 ± 1.50	2.69 ± 1.34	3.41 ± 1.26	3.73 ± 1.49	4.32 ± 1.36	119.69	< 0.001

### Area under ROC curve

**Figure 2:**
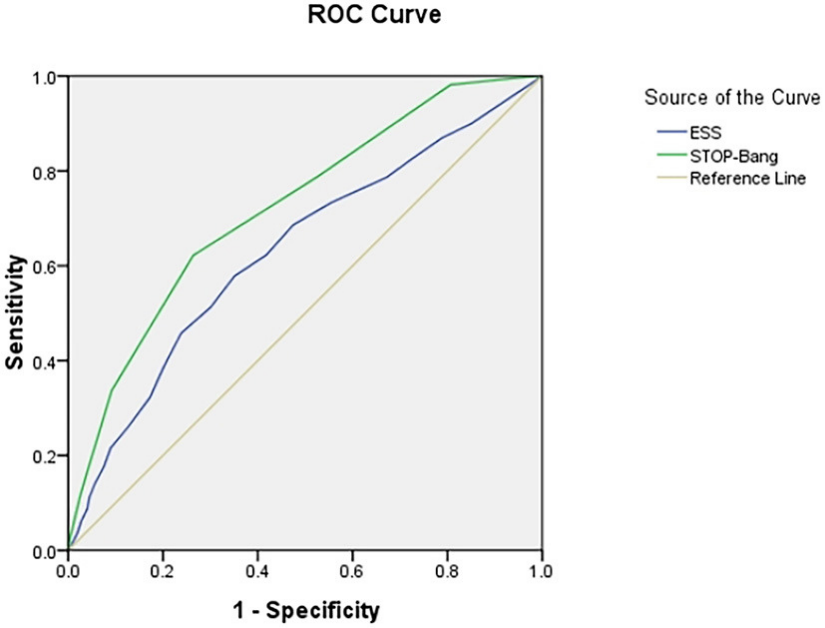
ROC curve of the ESS and STOP-Bang at AHI cutoff of ≥5 events/h (At an AHI cutoff of ≥5 events/h, the diagnostic performance of the STOP-Bang is better than the ESS. AHI, apnea-hypopnea index; ESS, Epworth Sleepiness Scale; STOP-Bang, STOP-Bang questionnaire; ROC, receiver operating characteristic).

**Figure 3:**
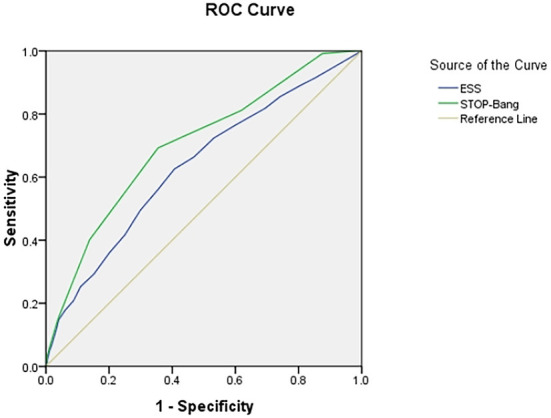
ROC curve of the ESS and STOP-Bang at AHI cutoff of ≥15 events/h (At an AHI cutoff of ≥15 events/h, the diagnostic performance of the STOP-Bang is better than the ESS. AHI, apnea-hypopnea index; ESS, Epworth Sleepiness Scale; STOP-Bang, STOP-Bang questionnaire; ROC, receiver operating characteristic).

**Figure 4:**
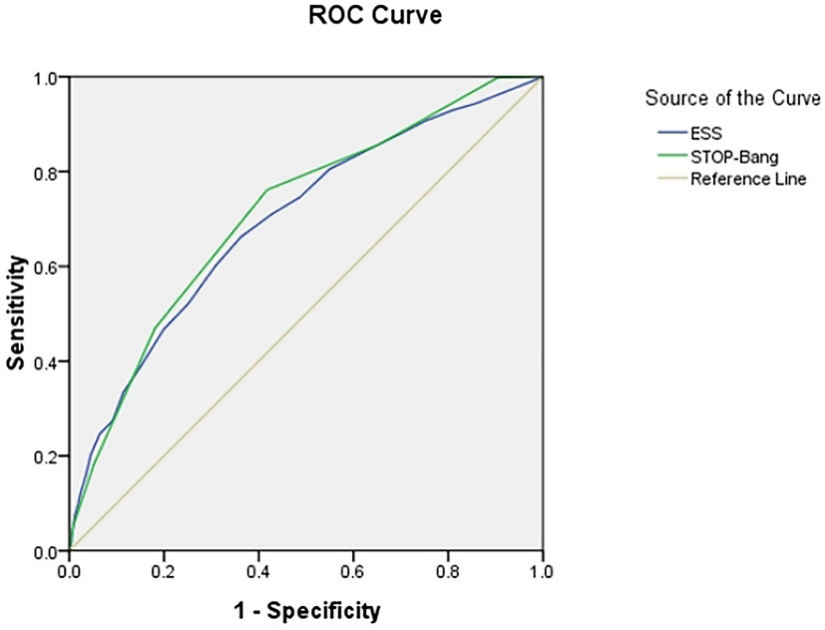
ROC curve of the ESS and STOP-Bang at AHI cutoff of ≥30 events/h (At an AHI cutoff of ≥30 events/h, the diagnostic performance of the STOP-Bang is better than the ESS. AHI, apnea-hypopnea index; ESS, Epworth Sleepiness Scale; STOP-Bang, STOP-Bang questionnaire; ROC, receiver operating characteristic).

The area under the curve (AUC) of the two scales was compared using AHI cutoffs of 5, 10, 15, 20, 25, and 30 events/h, respectively: STOP-Bang was 0.724, 0.704, 0.703, 0.702, 0.708, and 0.712, and ESS was 0.632, 0.618, 0.634, 0.653, 0.686, and 0.695. It was found that the AUC of STOP-Bang was higher than that of ESS (Table 2; Figures 2–4).

**Table 2:** The area under the receiver operating curve of various scales.

**AHI**	**ESS**	**STOP-Bang**
≥5	0.632 (0.603–0.661)	0.724 (0.697–0.751)
≥10	0.618 (0.591–0.644)	0.704 (0.679–0.729)
≥15	0.634 (0.607–0.660)	0.703 (0.679–0.728)
≥20	0.653 (0.626–0.680)	0.702 (0.677–0.728)
≥25	0.686 (0.658–0.713)	0.708 (0.682–0.735)
≥30	0.695 (0.667–0.723)	0.712 (0.686–0.739)

### Sensitivity and specificity of STOP-Bang

Using the STOP-Bang score of three as the cutoff, the sensitivity and specificity of STOP-Bang for OSA, moderate to severe OSA, and severe OSA were 0.788, 0.812, and 0.857, and 0.474, 0.381 and 0.348, respectively (Table 3).

**Table 3:** The scale predictors of each group patients [percentage (95%CI)].

**Scale**	**Sensitivity**	**Specificity**	**PPV**	**NPV**	**DOR**
AHI≥5 events/h					
ESS	0.455 (0.426–0.483)	0.762 (0.723–0.800)	0.830 (0.801–0.859)	0.353 (0.324–0.383)	2.665
STOP-Bang	0.788 (0.765–0.811)	0.474 (0.429–0.520)	0.793 (0.770–0.816)	0.467 (0.422–0.511)	3.349
AHI≥15 events/h					
ESS	0.492 (0.458–0.526)	0.702 (0.671–0.732)	0.616 (0.578–0.653)	0.587 (0.557–0.618)	2.279
STOP-Bang	0.812 (0.785–0.838)	0.381 (0.348–0.414)	0.560 (0.532–0.588)	0.676 (0.634–0.718)	2.651
AHI≥30 events/h					
ESS	0.592 (0.550–0.635)	0.693 (0.667–0.720)	0.454 (0.416–0.492)	0.798 (0.773–0.823)	3.289
STOP-Bang	0.857 (0.826–0.887)	0.348 (0.320–0.375)	0.361 (0.334–0.389)	0.849 (0.817–0.881)	3.189

### Specificity of STOP-Bang combined with ESS

The specificity of STOP-Bang (3) for OSA, moderate to severe OSA, and severe OSA was 0.474 (0.429–0.520), 0.381 (0.348–0.414) and 0.348 (0.320–0.375). When combined with ESS, the specificity increased to 0.668 (0.609–0.727), 0.598 (0.556–0.640) and 0.592 (0.557–0.627) (Table 4).

**Table 4:** Compare STOP-Bang combine ESS with STOP-Bang the scale predictors of each group patients [percentage (95%CI)].

**Scale**	**Sensitivity**	**Specificity**	**PPV**	**NPV**
AHI≥5 events/h				
STOP-Bang	0.788 (0.765–0.811)	0.474 (0.429–0.520)	0.793 (0.770–0.816)	0.467 (0.422–0.511)
STOP-Bang combine ESS	0.550 (0.518–0.581)	0.668 (0.609–0.727)	0.864 (0.836–0.891)	0.279 (0.243–0.315)
AHI≥15 events/h				
STOP-Bang	0.812 (0.785–0.838)	0.381 (0.348–0.414)	0.560 (0.532–0.588)	0.676 (0.634–0.718)
STOP-Bang combine ESS	0.585 (0.548–0.623)	0.598 (0.556–0.640)	0.650 (0.611–0.688)	0.531 (0.491–0.572)
AHI≥30events/h				
STOP-Bang	0.857 (0.826–0.887)	0.348 (0.320–0.375)	0.361 (0.334–0.389)	0.849 (0.817–0.881)
STOP-Bang combine ESS	0.675 (0.631–0.719)	0.592 (0.557–0.627)	0.483 (0.443–0.523)	0.763 (0.729–0.797)

### Two-step screening procedure screens for OSA risk

According to the above analysis, a two-step screening method can be formed. In the first step, STOP-Bang was used to screen all 1,671 patients; the risk of OSA, moderate to severe OSA, and severe OSA in patients with scores lower than 3 was 0.53 (0.49–0.58), 0.32 (0.28–0.37) and 0.15 (0.12–0.18), indicating that these patients were less likely to have OSA.

**Figure 5:**
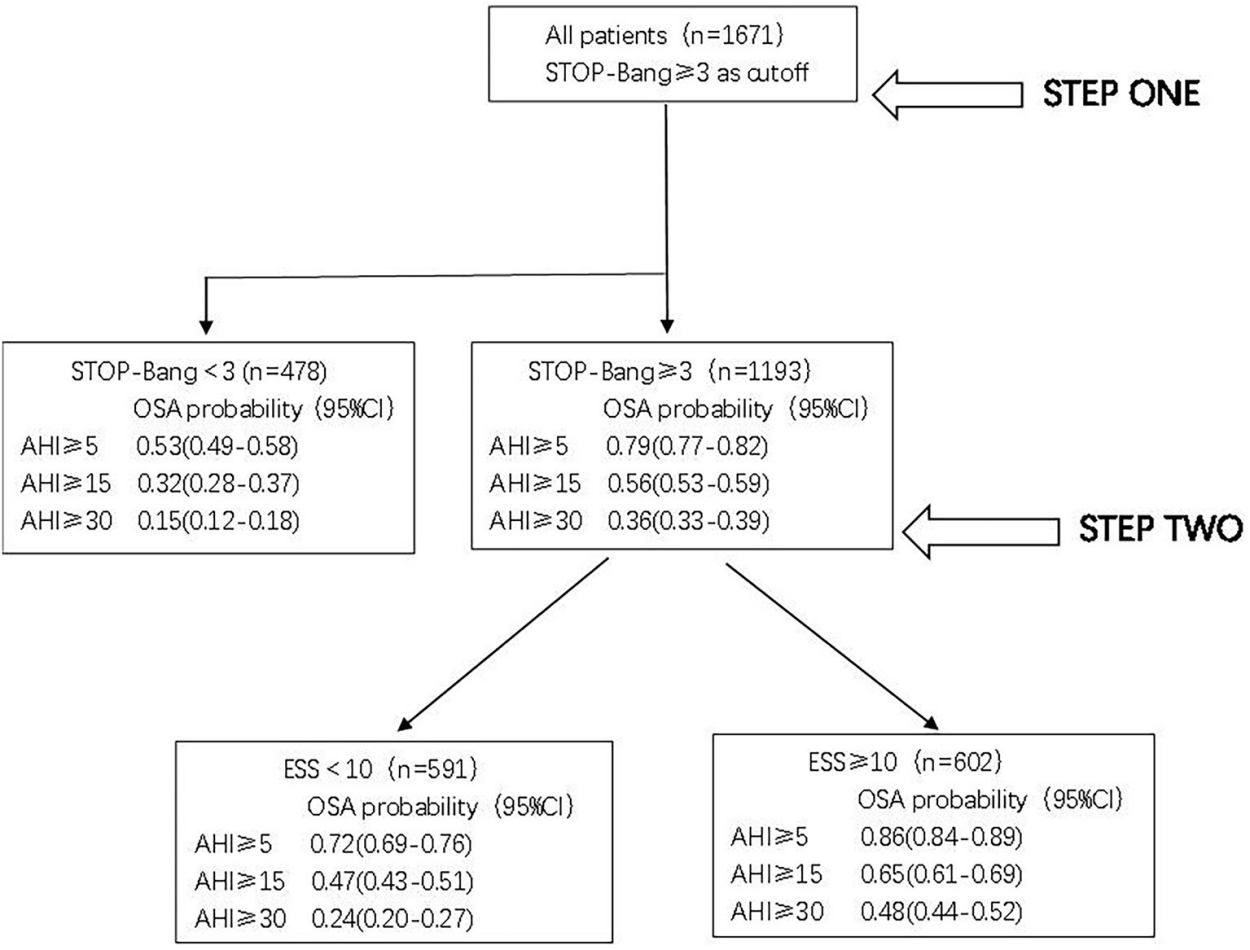
Screening strategy flowchart.

For 1,193 patients (STOP-Bang score ≥3), the risk of OSA was further evaluated by ESS. For patients with an ESS score ≥10, the risk of OSA, moderate to severe OSA, and severe OSA was 0.86 (0.84–0.89), 0.65 (0.61–0.69) and 0.48 (0.44–0.52). The risk was significantly higher than that of patients with ESS < 10; in particular, the risk of severe OSA was as much as 2.0 times higher (Figure 5).

## Discussion

In this study, 1,201 of the 1,671 patients suspected of OSA were confirmed, and the proportion of men was far higher than that of women, in line with the epidemiological characteristics of OSAHS. Scholars all over the world have developed a variety of OSA screening tools, among which a large number of scales are complex, requiring the use of computers and complicated mathematical calculations, making it difficult to promote the use of such tools in clinical practice (16–18). STOP-Bang is a relatively new questionnaire used for screening OSA. In this study, the STOP-Bang questionnaire showed an increasing trend with the aggravation of OSA, the comparison between the normal group and mild, moderate and severe OSA groups was statistically significant, and differences among OSA groups were also statistically significant. Similarly, these differences were reflected in ESS. However, there was no statistical difference between the mild and moderate OSA groups, suggesting that STOP-Bang is better than ESS at distinguishing severe OSA. As can be seen from the AUC, STOP-Bang was higher than ESS for OSA, moderate to severe OSA, and severe OSA, showing good predictive value for OSA patients. The results of this study are similar to those of other studies (19, 20), which show that the STOP-Bang questionnaire is a simple, effective and easy tool for risk assessment in patients with suspected OSA.

Diagnostic odds ratio (DOR) was applied in the meta-analysis to compare the accuracy of various prediction models and questionnaires for sleep-disordered breathing (21). In this study, the DOR values of STOP-Bang for OSA, moderate to severe OSA, and severe OSA were 3.349, 2.651, and 3.189 respectively, and the DOR values of ESS for OSA, moderate to severe OSA, and severe OSA were 2.665, 2.279, and 3.289 respectively. It can be seen that the DOR values of STOP-Bang in diagnosing OSA and moderate severe OSA are higher than those of ESS, while the DOR value of STOP-Bang in diagnosing severe OSA is similar to that of ESS. Therefore, STOP-Bang has better predictive value for OSA than ESS.

The lack of awareness of OSA among the public and health professionals leads to a failure in the timely diagnosis of OSA patients, and studies have found that the vast majority (>80%) of patients with moderate to severe OSA remain undiagnosed (22). Untreated OSA patients are at increased risk for metabolic syndrome, cardiovascular disease and impaired neurocognitive function and mental health (23–26), and the disease significantly reduces the quality of life of patients (27, 28) and even contributes to their premature death (29–31). The adverse health effects of OSA can significantly increase economic costs (32, 33), while the treatment of OSA can bring economic benefits (34). Although PSG is the gold standard for diagnosing OSA, it is time-consuming and expensive, and has long waiting times, making timely diagnosis difficult to achieve. Tools for screening patients at high risk for OSA are becoming increasingly important in order to identify patients with OSA early and schedule further diagnosis and treatment.

The results of this study provide a simple and effective program for screening suspected OSA patients. We recommend a two-step screening. The first step is preliminary screening with the STOP-Bang questionnaire. Those with scores lower than three have a low risk of OSA, but its low specificity can easily cause a high false positive rate, so it is necessary to conduct the second step screening for those with scores of three or above.

The ESS score is mainly based on the patients' own cognitive score, which is completely subjective, while the STOP-Bang questionnaire is mainly based on objective factors. The combination of the two scales can complement each other to improve the predictive value of OSA. In this study, when combined with ESS, the specificity of the STOP-Bang questionnaire in predicting OSA, moderate to severe OSA, and severe OSA in patients was 0.668, 0.598, and 0.592. It can be seen that the specificity of the STOP-Bang questionnaire combined with ESS in predicting OSA patients can be significantly improved. Therefore, we suggest that the second step of screening should be carried out in combination with ESS for people with a STOP-Bang questionnaire of three or more. For patients at high risk for OSA, we suggest a PSG examination followed by stratified management according to the examination results, including behavior adjustment, weight loss, drugs, continuous positive airway pressure ventilation, oral appliances, surgery and other individualized treatments. With the deepening of public understanding of OSA and the formation and efforts of multidisciplinary teams, the layered management of OSA is becoming increasingly important, and the rational diagnosis and treatment of OSA can be expected in the future.

Like many studies, this study has several shortcomings. The single-center cohort study mainly includes subjects from Guangzhou, but as the National Respiratory Medicine Center, our unit accepts a large number of research subjects from all over the country and should represent the Chinese population to a certain extent; and the members of the normal and severe OSAHS groups were younger than those of the mild and moderate groups, which may have had a certain impact on the results.

In conclusion, combining ESS with the STOP-Bang score improves its specificity at the cost of reducing its sensitivity in predicting OSA. As such, we recommend a two-step screening process for suspected OSA patients: the initial screening using the highly sensitive STOP-Bang score (three points), and then combining it with ESS to improve the specificity. This screening approach can assist doctors in conducting stratified management according to the OSA risk levels of patients, identifying high-risk patients and having them undergo PSG examination as soon as possible, and carrying out early intervention for patients with a definite diagnosis, thereby minimizing the harm caused by OSA.

## Data availability statement

The raw data supporting the conclusions of this article will be made available by the authors, without undue reservation.

## Ethics statement

The studies involving human participants were reviewed and approved by Medical Ethics Committee of the First Affiliated Hospital of Guangzhou Medical University. Written informed consent for participation was not required for this study in accordance with the National Legislation and the Institutional requirements. Written informed consent was not obtained from the individual(s) for the publication of any potentially identifiable images or data included in this article.

## Author contributions

YZ, ZZh, CL, MC, and CW are the guarantor of the manuscript and take responsibility for the content of this manuscript. YZ, ZZh, RC, and XC contributed to the design of the study. JZ, JL, and XO were involved in the data analysis. ZZo, XO, and RC contributed to the acquisition of primary data. JL and RC wrote the initial draft of the manuscript. ZZh, RC, JD, and XC contributed significantly to the revision of the manuscript. All authors contributed to the article and approved the submitted version.

## Funding

This study was funded by the Natural Science Foundation of Guangdong Province (2021A1515011373).

## Conflict of interest

The authors declare that the research was conducted in the absence of any commercial or financial relationships that could be construed as a potential conflict of interest.

## Publisher's note

All claims expressed in this article are solely those of the authors and do not necessarily represent those of their affiliated organizations, or those of the publisher, the editors and the reviewers. Any product that may be evaluated in this article, or claim that may be made by its manufacturer, is not guaranteed or endorsed by the publisher.
